# A double-blind randomized control trial of transcranial direct current stimulation in post-stroke fatigue

**DOI:** 10.3389/fneur.2026.1658764

**Published:** 2026-05-07

**Authors:** Wai Kwong Tang, Hanna Lu, Thomas Wai Hong Leung, Kenneth Nai Kuen Fong, Selina Kit Yi Chan, Vivien Wei Jun Liew

**Affiliations:** 1 Department of Psychiatry, The Chinese University of Hong Kong, Hong Kong, Hong Kong SAR, China; 2 Department of Medicine and Therapeutics, The Chinese University of Hong Kong, Hong Kong, Hong Kong SAR, China; 3 Department of Rehabilitation Sciences, The Hong Kong Polytechnic University, Hong Kong, Hong Kong SAR, China

**Keywords:** post-stroke fatigue (PSF), randomized controlled trial (RCT), rehabilitation, stroke, transcranial direct current stimulation (tDCS)

## Abstract

**Rationale:**

Post-stroke fatigue (PSF) is an issue among stroke survivors that often impedes their rehabilitation progress. Treating PSF is challenging, and pharmacological interventions often prove ineffective.

**Aims:**

The aim of this study was to examine the effect of tDCS on PSF.

**Sample size:**

Thirty-four participants aged 30 to 80 with chronic stroke were recruited and randomly assigned to one of two groups, with 17 participants in each group.

**Methods and design:**

This study was a double-blind randomized controlled trial. The sham group received sham tDCS, while the treatment group received active tDCS. The active tDCS treatment consisted of applying a constant 2-mA current through a 5 cm × 5 cm anodal electrode placed over the C3 or C4 positions (motor cortex) of the contralateral hemisphere of the scalp, with the cathodal electrode placed on the ipsilateral arm. The participants received two 20-min sessions of this treatment, separated by a 10-min interval, each day for 5 consecutive days. Sham tDCS involved the same setup but with only 30 s of constant current at the beginning and end of each 20-min session. Follow-up assessments were conducted over an 8-week period. The effects of tDCS were calibrated using an analysis of covariance approach, with baseline Modified Fatigue Impact Scale (MFIS) scores, age, and education as covariates. The inclusion criteria were (1) either sex; (2) age 30–80 years; (3) prior stroke diagnosis verified through brain imaging (computed tomography scan/magnetic resonance imaging); (4) Chinese ethnicity and Cantonese proficiency; (5) willingness and ability to provide informed consent; (6) presence of PSF (Fatigue Severity Scale score ≥ 4.0); and (7) at least 6 months post-stroke.

**Study outcome:**

The primary outcome was the change in fatigue severity, assessed using the MFIS.

**Results:**

One participant in the sham group dropped out. After the intervention, no significant changes were observed in MFIS scores at any of the follow-up timepoints (*p* > 0.05).

**Conclusion:**

We found no evidence that the use of tDCS improves PSF. Further research is needed to explore the potential of this non-invasive brain stimulation method for the treatment of PSF.

**Clinical trial registration:**

https://clinicaltrials.gov/, identifier NCT04238260; https://www.chictr.org.cn/, identifier ChiCTR2100052515.

## Introduction

Fatigue is defined as the “subjective lack of physical or mental energy to carry out usual and desired activities as perceived by the patient” (1). Patients experiencing fatigue often feel an overwhelming sense of tiredness, exhaustion, or lack of energy during or following mental or physical activities. Fatigue is frequently observed as a symptom in neurological disorders (2).

Post-stroke fatigue (PSF) is a prevalent and long-lasting issue (3), occurring in 23 to 85% of cases (3–9). For many patients who recover well after stroke, PSF remains their sole major disability (10). Up to 40% of stroke patients report PSF as the most challenging consequence of their stroke (3). PSF can impede rehabilitation efforts (5) and is associated with decreased functional independence, a higher likelihood of institutionalization, cognitive impairments, reduced quality of life (9), and increased mortality (11, 12). Clinical factors linked to PSF include female sex, older age, and a history of previous stroke (11). Additional causes of PSF may include sleep apnea (13) and depression (3, 14). PSF is a multifaceted condition with numerous contributing factors, many of which remain poorly understood.

Potential pharmacological options for PSF, including antidepressants, stimulants, vitamin D, and wakefulness-promoting agents such as modafinil, have not shown significant effectiveness (15, 16). Likewise, the efficacy of non-pharmacological approaches, such as low-intensity exercise, has yet to be conclusively demonstrated (17).

Transcranial direct current stimulation (tDCS), which involves applying a low-intensity current to the cortex, can modulate sensory, cognitive, motor, and behavioral functions (18). tDCS influences neuronal activities and produces protracted after-effects on brain function, and is a cost-effective and portable method for neuromodulation (19).

There is growing interest in tDCS as a therapy to support stroke recovery. Clinical trials have indicated that tDCS may be effective in treating various motor (20) and non-motor impairments in stroke patients. tDCS appears to be more beneficial for stroke patients with mild-to-moderate impairments than for those with severe deficits (21).

A recent systematic review of the effects of tDCS on fatigue in patients with neurological disorders found that all of the 42 included studies had used anodal tDCS (22). PSF is associated with reduced motor cortical excitability in the affected hemisphere (23). Relatedly, a recent review of functional neuroimaging studies found that the development of PSF may be partly driven by altered cortical excitability (24). Moreover, it was found that anodal stimulation increases cortical excitability in the stroke-affected hemisphere. This reactivation of the perilesional cortical area appears to be the mechanism whereby anodal tDCS alleviates motor deficits in patients with stroke (25). Hence, anodal stimulation may increase cortical excitability and reduce PSF symptoms.

An open-label trial administering two tDCS sessions per week for 5–6 weeks in 10 stroke patients found no improvement in PSF (26). In contrast, a recent clinical trial involving 30 PSF patients demonstrated that a single session of anodal tDCS applied to the bilateral primary motor cortex reduced PSF symptoms for up to seven days compared with sham stimulation (23). One randomized controlled trial (RCT) involving 74 chronic stroke patients with fatigue revealed no additional benefit of six tDCS sessions (27). However, another RCT involving 60 stroke patients reported that six tDCS sessions per week over 4 weeks led to a reduction in fatigue (28). Currently, a trial is underway testing six tDCS sessions in 100 patients with PSF (29).

A recent review identified 42 studies investigating the effects of tDCS on fatigue in patients with neurological disorders, involving a total of 994 participants. Improvements in fatigue scores were reported in 36 of the 42 studies (85.7%) following tDCS treatment. Side-effects associated with tDCS are generally mild (22). Hence, tDCS has emerged as a simple, low-cost, portable, non-invasive, and safe method with promising potential for reducing fatigue symptoms and alleviating PSF. However, the above-mentioned recent review (22) identified only five studies that targeted PSF patients. Of these five studies, two examined long-term effects (i.e., over 1–2 months), one examined the effects of bilateral tDCS (in a single session), and none used multidimensional measures of fatigue symptoms. Hence, tDCS for PSF management remains underexplored. Consequently, the aim of this study was to assess the long-term impact of consecutive bilateral tDCS on the physical, cognitive, and psychosocial symptoms of PSF. The study hypothesis was that active tDCS would be more effective than sham stimulation.

## Materials and methods

### Design

**Figure 1:**
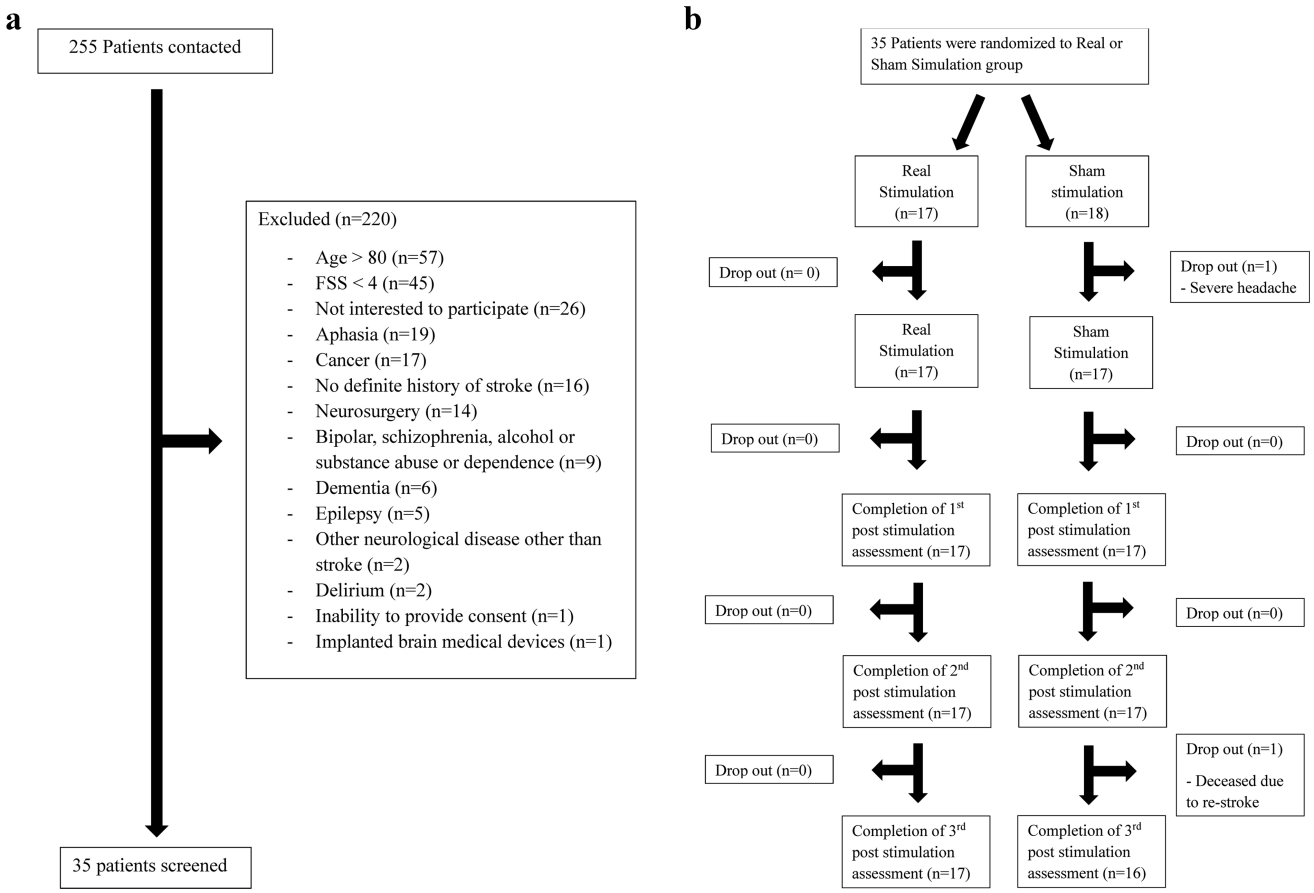
**(a)** Study flow diagram (recruitment). **(b)** Study flow diagram (treatment group allocation).

A double-blind RCT of stroke survivors was conducted (Figures 1a,b).

### Patient population

Patients were recruited from multiple sources, including the Neurology Unit of the Prince of Wales Hospital, the rehabilitation wards of the Geriatric Day Hospital of Shatin Hospital, the Lek Yuen General Out-Patient Clinic, and the Geriatric Day Hospitals of the Tai Po Hospital and Alice Ho Miu Ling Nethersole Hospital. Additionally, recruitment efforts were extended to self-help groups and via word-of-mouth. A research assistant (RA) made weekly visits to all hospitals to identify all eligible patients and secure their written consent. For recruitment from community settings, prior to the intervention, phone calls were made to filter out those who were ineligible.

### Inclusion and exclusion criteria

The inclusion criteria were (1) either sex; (2) an age of 30–80; (3) a prior stroke diagnosis verified through brain imaging, such as a computed tomography scan or magnetic resonance imaging; (4) of Chinese ethnicity and able to speak Cantonese; (5) willingness and ability to provide informed consent; (6) the presence of PSF (a Fatigue Severity Scale [FSS] score of 4.0 or more) (30–33); and (7) at least 6 months post-stroke. The FSS cut-off score of 4.0 was selected as it is the most frequently used threshold. A recent systematic review of 31 studies examining the prevalence of PSF using the FSS found that all studies applied a cut-off score of 4.0 or higher (8).

The exclusion criteria were (1) acute or subacute stroke (≤6 months after onset); (2) the presence of schizophrenia, bipolar affective disorder, and/or substance/alcohol dependence/abuse; (3) a history of any neurological disorder (except stroke); (4) a history of traumatic brain injury or cancer; (5) ongoing pregnancy; (6) a history of neurosurgery; (7) a Mini-Mental State Examination (MMSE) score of less than 19 (34); (8) presence of aphasia (35); (9) current use of hypnotic drugs or other medications that may induce fatigue; (10) current use of antiepileptic drugs or other medications that can reduce the effectiveness of tDCS; (11) ongoing or previous application of tDCS; or (12) contraindications to tDCS, such as skin injury at the stimulation site, the presence of a pacemaker, metal implants in the head, or implanted medical devices in the brain. The above-mentioned criteria were assessed through examination of the patients’ medical records.

### Study incentives

All participants received compensation of HKD500 upon the completion of five tDCS treatment sessions and three follow-up assessments.

### Measurement overview

**Figure 2:**
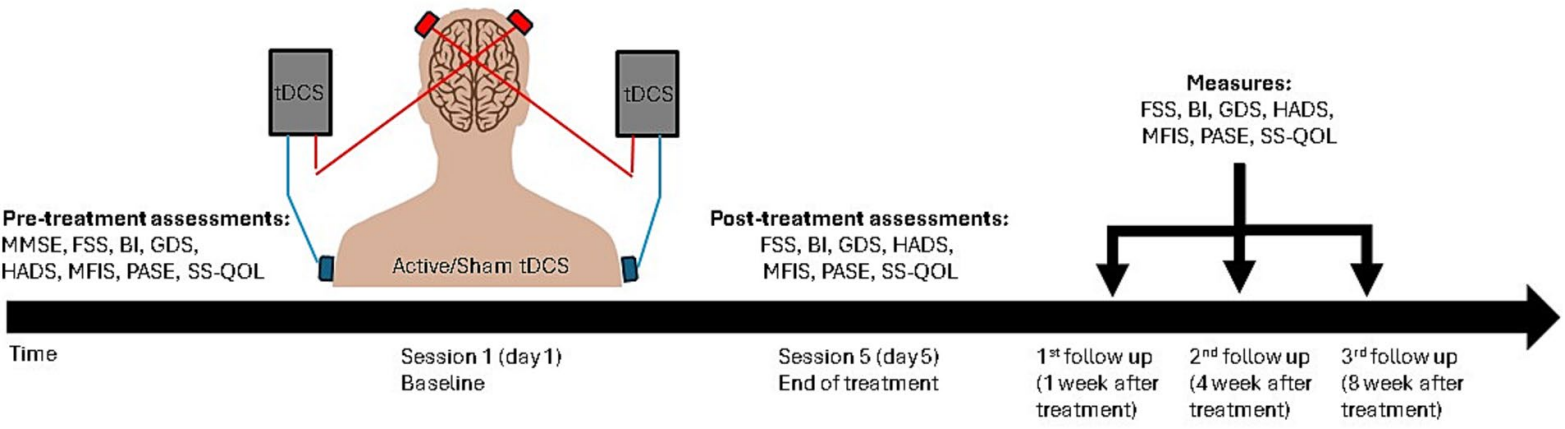
Overview of the study design and procedures conducted during each session across five distinct time points.

The data collection schedule is detailed in Table 1 and Figure 2. The number of patients excluded, along with the reasons for their exclusion, was documented. Data on the sex, age, level of education, risk factors for vascular diseases (e.g., smoking, hyperlipidemia, diabetes mellitus, and hypertension), and date of stroke onset were collected from all of the participants.

**Table 1:** Data collection schedule.

Study period	Screening	Treatment	EOT	FUV
Visit	1	2–6	7	8–10
Weeks after randomisation	-2	1	1	2, 3, 5
Review of inclusion / exclusion criteria	X			
Informed consent	X			
Demographics, vascular risk factors, stroke characteristics, previous treatment of PSF	X			
Montreal cognitive assessment	X			
MFIS, FFS, PSAE, BI, GDS, HADSA, SSQoL, MMSE	X		X	X
tDCS		X		
Randomisation	X			
tDCS adverse effects questionnaire		X	X	
Experience, preferences, concerns and belief on tDCS questionnaire			X	

Screen denotes screening visit.

EOT denotes end of treatment.

FUV denotes follow up visits.

### Baseline measures

An RA evaluated the participants’ PSF using the validated Chinese version of the FSS (36, 37) and the Modified Fatigue Impact Scale (MFIS) (38–40). The FSS is the most commonly used instrument for assessing PSF (41–43). It includes nine items rated on a 7-point Likert scale (e.g., “I am easily fatigued,” “My motivation is lower when I am fatigued than when I am not,” and “Exercise brings on my fatigue”). A higher item score reflects greater fatigue, and the average of all item scores provides a total fatigue score. In evaluating the FSS among patients with neurological disorders, Cronbach’s *α* ranged from 0.90 to 0.94, the intraclass correlation coefficient ranged from 0.73 to 0.93, and the correlation coefficients with other fatigue scales ranged from 0.62 to 0.84, demonstrating that the FSS possesses good internal consistency, reliability, and convergent validity in this population (42).

The MFIS is conducted through a 5–10-min interview that evaluates the impact of fatigue on an individual’s daily psychosocial, cognitive, and physical functioning. The MFIS consists of 21 items scored from 0 to 4, corresponding to frequency categories of “never,” “seldom,” “sometimes,” “often,” and “always.” It generates sub-scores for cognitive functioning (0–36), physical functioning (0–40), and psychosocial functioning (0–8), which are summed to produce a total score (0–84). Higher scores indicate greater fatigue severity. The MFIS has been utilized to assess PSF (44) and to evaluate outcomes in clinical trials of fatigue interventions (45), including tDCS (46, 47). By assessing the physical, cognitive, and psychosocial effects of fatigue, the MFIS provides a more complete picture than the unidimensional FSS.

Anxiety and depressive symptoms are common comorbidities and correlates of PSF, while physical activity, disability, and quality of life are important functional outcomes that reflect one’s daily functioning and well-being. Accordingly, an RA employed several assessment tools to evaluate the participants: the Physical Activity Scale for the Elderly (PASE) to measure their physical activity levels (48); the Barthel Index (BI) to assess their disability (49); the MMSE to assess their global cognitive function; the anxiety subscale of the Chinese version of the Hospital Anxiety Depression Scale (HADSA) to evaluate their anxiety symptoms; the Chinese version of the Geriatric Depression Scale (GDS) to evaluate their depressive symptoms; and the Chinese version of the Stroke-Specific Quality of Life (SSQoL) scale to assess their health-related quality of life.

The Chinese version of the SSQoL scale (50, 51) is designed to assess quality of life in stroke survivors. This self-report questionnaire includes 49 items across 12 domains: family roles (three items), energy (three items), mobility (six items), language (five items), mood (five items), personality (three items), social roles (five items), self-care (five items), upper-extremity function (five items), thinking (three items), work/productivity (three items), and vision (three items). Each item is rated on a 5-point Likert scale ranging from “completely true” to “not true at all,” with higher scores indicating better quality of life. Consequently, the Chinese version of the SSQoL scale provides both domain-specific and total scores. Previous studies have demonstrated its excellent internal consistency, intertest reliability, and test–retest reliability (50).

The anxiety subscale of the Chinese version of the HADSA was used to assess the participants’ anxiety severity *over the past week* (52, 53). This subscale comprises seven items, such as “I feel keyed up, on edge,” “I have been worrying a lot,” and “I have been irritable.” Each item is rated on a 4-point Likert scale, ranging from 0 (“not at all”) to 3 (“most or all of the time”), and items 4 and 5 are reverse coded. The total possible score ranges from 0 to 21, with higher scores indicating more severe anxiety than lower scores. The Chinese version of the HADSA has been validated extensively in various patient populations in China, demonstrating good construct validity and psychometric properties (53–56). Moreover, its reliability estimates in Chinese samples have shown acceptable to good internal consistency, with Cronbach’s alpha values typically ranging from 0.68 to 0.93 (53, 55, 57). Furthermore, it has been employed in studies focused on stroke patients in Hong Kong, underscoring its relevance and applicability to this population (58, 59).

The Chinese version of the GDS (60, 61) comprises 15 items with yes/no responses (0 = “yes,” 1 = “no”) reflecting feelings *over the past week* (e.g., “Have you dropped many of your activities and interests?”; “Do you feel that your life is empty?”; and “Do you often get bored?”). Items 1, 5, 7, 11, and 13 are reverse coded. Total possible scores range from 0 to 15, with higher scores indicating more severe depressive symptoms than lower scores. The Chinese version of the GDS has demonstrated good validity and reliability in the Chinese population (60, 62, 63) and in stroke populations in Hong Kong (61, 64). Cronbach’s alpha values for the scale in various Chinese stroke populations have ranged from 0.74 to 0.89, indicating good internal consistency (61, 63, 64).

The Chinese version of the PASE (48, 65) comprises questions on the frequency and duration of respondents’ leisure activities (six questions on, e.g., watching TV or a taking a stroll outside the home), household activities (three questions on, e.g., performing light housework such as dishwashing, or heavy chores such as mopping the floor), and work-related activities (one question on working for pay/as a volunteer) *over the past week*. The frequency of an activity is rated on a 4-point Likert scale, ranging from 0 (“never”) to 3 (“always”), while the duration of an activity is rated on a 4-point Likert scale, ranging from 1 (“less than 1 hour”) to 4 (“more than 4 hours”). These responses are converted into hours per day or days per week using a conversion table and then multiplied by predefined activity weights specific to each activity category. The weighted values for all activities are summed to generate a total score that can range from 0 to over 400, with higher scores indicating higher physical activity levels. The Chinese version of the PASE has been well validated and found to have excellent psychometric properties, including high test–retest reliability (intraclass correlation coefficients of 0.79–0.81) in Chinese older adult populations (57, 65).

The Chinese version of the BI (49, 57) comprises 10 items measuring functional independence across activities such as feeding, bathing, grooming, dressing, bowel and bladder control, toilet use, transfers from bed or chair, walking, and stair climbing. Each activity is scored based on the level of assistance required, and the total possible scores range from 0 to 100, with higher scores indicate greater independence and functional ability. The Chinese version of the BI has been widely used in studies involving Chinese populations and has demonstrated excellent validity and reliability, with a Cronbach’s alpha range of approximately 0.90 to 0.95 (66, 67). Additionally, a recent study confirmed its robustness for evaluating stroke survivors in China (68), and it was validated for use in stroke rehabilitation settings in Hong Kong (57), where it exhibited high internal consistency, with a Cronbach’s alpha of 0.93. These findings indicate that the Chinese version of the BI is a reliable tool for the clinical assessment of post-stroke functional outcomes in Hong Kong populations.

### Randomization and blinding procedures

A statistician used a block randomization method to generate a concealed randomization list.

Paper cards indicating “sham” or “active” were prepared and placed inside opaque black envelopes by the principal investigator. Next, an envelope was randomly selected to assign the intervention for each participant, ensuring randomization and the blinding of participants to their group assignment. Two RAs were involved: one RA administered the tDCS and thus was aware of the group assignment, while the other RA conducted all the outcome assessments and was blinded to the group assignment. Each group comprised 17 participants (the sham tDCS and active tDCS groups). Upon enrolling each participant, the RA administering the tDCS was informed of the participant’s treatment assignment.

### Intervention

**Figure 3:**
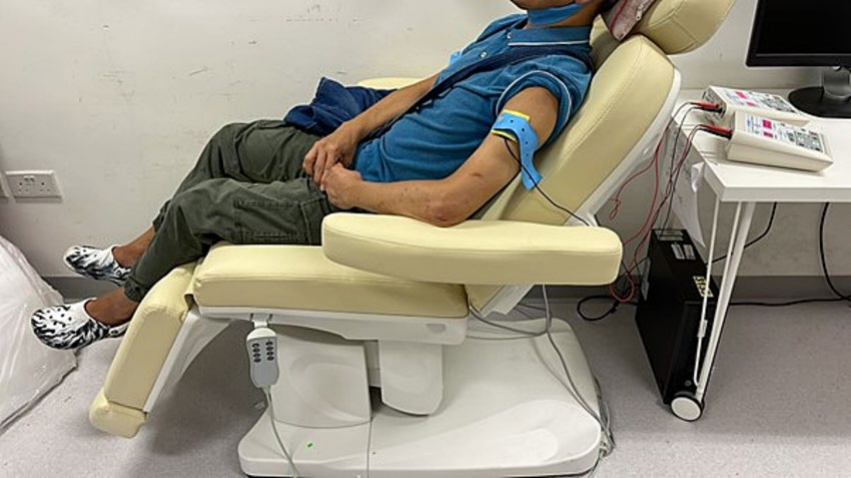
Set up of the tDCS treatment.

A trained RA conducted the tDCS sessions at Shatin Hospital. During the stimulation, the participants reclined comfortably on a chair with their eyes closed. The tDCS was administered using two battery-powered constant-current stimulators (1×1 Clinical Trials Device; Soterix Medical, Woodbridge, NJ, USA) via a 5 cm × 5 cm conductive-rubber anodal electrode. The electrode was placed inside an EASYpad (Soterix Medical) containing sponge material, which was moistened with approximately 10–15 mL of saline. The EASYpad was checked every minute to determine whether additional saline was needed to prevent drying. Each participant was provided with a dedicated set of EASYpads for the entire treatment course to ensure good hygiene. Elastic straps were used to secure the EASYpad to the scalp. The anodal electrode was placed on the scalp over the C3 or C4 position (motor cortex) of the contralateral hemisphere according to the international electroencephalogram 10/20 system, while the cathodal electrode was placed on the ipsilateral arm (29) (Figure 3). A constant current of 2 mA (current density 0.08 mA/cm (2)) was applied to the anode using one of the constant-current stimulators in two 20-min sessions, separated by a 10-min break (23), administered daily for 5 consecutive days. Sham tDCS involved 30 s of constant current at the start and end of each 20-min session, such that all participants experienced the initial itching sensation at the beginning of the stimulation. The stimulation site and parameters were selected based on the following rationale. First, an association has been observed between increased fatigue scores and reduced functional connectivity of supplementary and primary motor areas (22), and prior findings have shown that stimulation of the motor cortex can alleviate fatigue in stroke patients and healthy individuals (23, 69). Second, the duration of stimulation per session was 20 min in all five published studies of the use of tDCS to treat PSF (22). Third, consecutive sessions of tDCS have been employed in earlier tDCS trials for PSF (27, 29).

### Safety measures

After the final stimulation session, we assessed adverse effects using a tDCS side-effects survey (26), which covers questions regarding the area, time, and degree of discomfort experienced during the tDCS procedure. In addition, we collected feedback from the participants after the final stimulation via a tDCS attitude-and-impressions survey, designed to determine participants’ attitudes toward and impressions of the feasibility, benefits, and barriers to the use of tDCS (26).

### Withdrawal criteria

The participants completed 10 tDCS sessions within 1 week. If a scheduled session was missed, it was rescheduled at a convenient time. Participants who did not complete all 10 sessions were considered dropouts. Additionally, participants were withdrawn from the study if they (1) revoked their consent for tDCS treatment and/or (2) experienced a severe adverse event causing significant distress, such as a seizure, skin burn, or blister. We continued to conduct follow-ups to perform outcome measures with participants who withdrew from the study whenever possible.

### Primary outcome measure

The MFIS was administered at baseline (T0), immediately after 10 treatment sessions (T1, 1 day post-final treatment), and at follow-up intervals of 1 week (T2), 4 weeks (T3), and 8 weeks (T4) thereafter (70), consistently at the same time of day. Both the participants and the RA administering the MFIS were blinded to the treatment allocation.

### Secondary outcome measures

The FSS, the Chinese versions of the GDS and the HADSA, the BI, and the PASE were administered at T0, T1, T2, T3, and T4 (70), consistently at the same time of day. The Chinese version of the SSQoL was administered at T0 and T4. The timing of assessments was adjusted as previously described to capture changes between time points. The participants’ attitudes toward and experiences with tDCS were collected at the final treatment using a brief questionnaire (26).

A Data Safety Monitoring Board (DSMB) comprising a psychiatrist and a tDCS clinical trial expert was established. The DSMB convened regularly to review adverse events, conduct interim analyses, and offer recommendations regarding the study’s safety and whether the study should be stopped or extended.

### Statistical analysis

All enrolled participants were included in the analysis. The overall MFIS score, along with physical, cognitive, and psychosocial sub-scores, were derived. Descriptive analyses were conducted on participants’ baseline characteristics, including age, sex, clinical characteristics, and scores from the MFIS, FSS, GDS, HADSA, PASE, BI, and SSQoL. Chi-square tests were used for categorical variables (e.g., sex), and Student’s t-tests for continuous variables (e.g., age) were utilized to compare baseline characteristics. For the primary outcome, analysis of covariance was performed on the MFIS overall and sub-scores, adjusting for baseline MFIS scores and other significantly different baseline characteristics, with group assignment as the factor. These analyses were repeated for the secondary outcomes, including the FSS, PASE, BI, GDS, HADSA, and SSQol scores. Safety analysis was descriptive and exploratory, focusing on key safety measures derived from the tDCS adverse events questionnaire, including the overall incidence and severity of side-effects. A significance level of 0.05 was applied throughout.

## Results

### Demographic and baseline data

Two hundred and fifty-five patients were contacted, of whom 220 were excluded for the following reasons: aged over 80 (*n* = 57), an FSS score < 4 (*n* = 45), aphasia (*n* = 19), cancer (*n* = 17), no definite history of stroke (*n* = 16), prior neurosurgery (*n* = 14), psychiatric disorder (*n* = 9), dementia (*n* = 6), epilepsy (*n* = 5), another neurological disease unrelated to stroke (*n* = 2), delirium (*n* = 2), inability to provide consent (*n* = 1), or an implanted medical device in the brain (*n* = 1) (Figure 1a). Thirty-five patients were enrolled, with 17 allocated to the active tDCS group and 18 allocated to the sham group. One participant in the sham group dropped out due to experiencing a side-effect (i.e., severe headache). Among those who completed the trial, 64.7% were recruited from self-help groups and 35.3% from hospitals. The active tDCS group was older (66.5 versus 57.3 years, *p* = 0.003) than the sham group and showed a trend toward a lower educational level (7.7 versus 10.1 years, *p* = 0.051). There were no significant differences between the two groups in terms of vascular risk factors, the duration of stroke, or baseline scores for fatigue, cognitive function, mood, or quality of life (Table 2). All participants in the active tDCS group completed all tDCS sessions and follow-up assessments. In the sham group, one participant received only 4 days of treatment, and two participants missed the T4 follow-up assessment (Figure 1b).

**Table 2:** Patient demographics and baseline clinical data for the real and sham stimulation groups.

Baseline characteristics	Active *(N = 17)*	Sham *(N = 17)*	*P^a^*
Age (years)	66.5 ± 7.5	57.3 ± 9.2	0.003
Gender (male/ female)	10/7 (58.8/41.2)	11/6 (64.7/35.3)	0.724^b^
Education years (years)	7.7 ± 4.1	10.1 ± 2.7	0.051
Smoker	4 (23.5)	6 (35.3)	0.452^b^
Hypertension	14 (82.4)	15 (88.2)	0.628^b^
Diabetes mellitus	5 (29.4)	5 (29.4)	1.000^b^
Hyperlipidaemia	9 (52.9)	12 (70.6)	0.290^b^
Time since stroke (years)	7.2 ± 10.3	5.5 ± 5.0	0.587
MMSE scores	24.9 ± 4.2	25.5 ± 4.5	0.725
FSS scores	49.2 ± 6.3	50.1 ± 6.3	0.665
MFIS scores	40.3 ± 16.3	38.3 ± 15.7	0.718
Physical	16.6 ± 6.4	17.8 ± 7.0	0.595
Cognitive	17.8 ± 8.4	15.2 ± 7.2	0.343
Psychosocial	3.5 ± 2.5	3.1 ± 2.5	0.635
PASE scores	86.1 ± 57.0	71.3 ± 60.9	0.469
BI scores	94.1 ± 10.2	95.6 ± 6.3	0.618
GDS scores	10.3 ± 3.5	8.5 ± 5.1	0.251
0–5: normal	2 (11.8)	5 (29.4)	0.203^b^
6–9: (minor depressive disorder)	4 (23.5)	4 (23.5)	1.000^b^
10–15 (major depressive disorder)	11 (64.7)	8 (47.1)	0.300^b^
HADSA scores	6.6 ± 4.5	4.7 ± 3.8	0.182
0–7: normal	10 (58.8)	13 (76.5)	0.271^b^
8–10: (possible anxiety)	4 (23.5)	2 (11.8)	0.368^b^
11–21:(probable anxiety)	3 (17.6)	2 (11.8)	0.628^b^
SSQoL scores	175.4 ± 39.0	180.1 ± 34.4	0.712

MMSE = Mini-mental State Examination, FFS = Fatigue Severity Scale, MFIS = Modified Fatigue Impact Scale, PASE = Physical Activity Scale for the Elderly, BI = Barthel Index, GDS = Geriatric Depression Scale, HADS = Hospital Anxiety and Depression Scale, SSQoL = Stroke Specific Quality of Life Scale.

^a^Independent T-test; ^b^Chi-square test.

### Primary outcome

**Figure 4:**
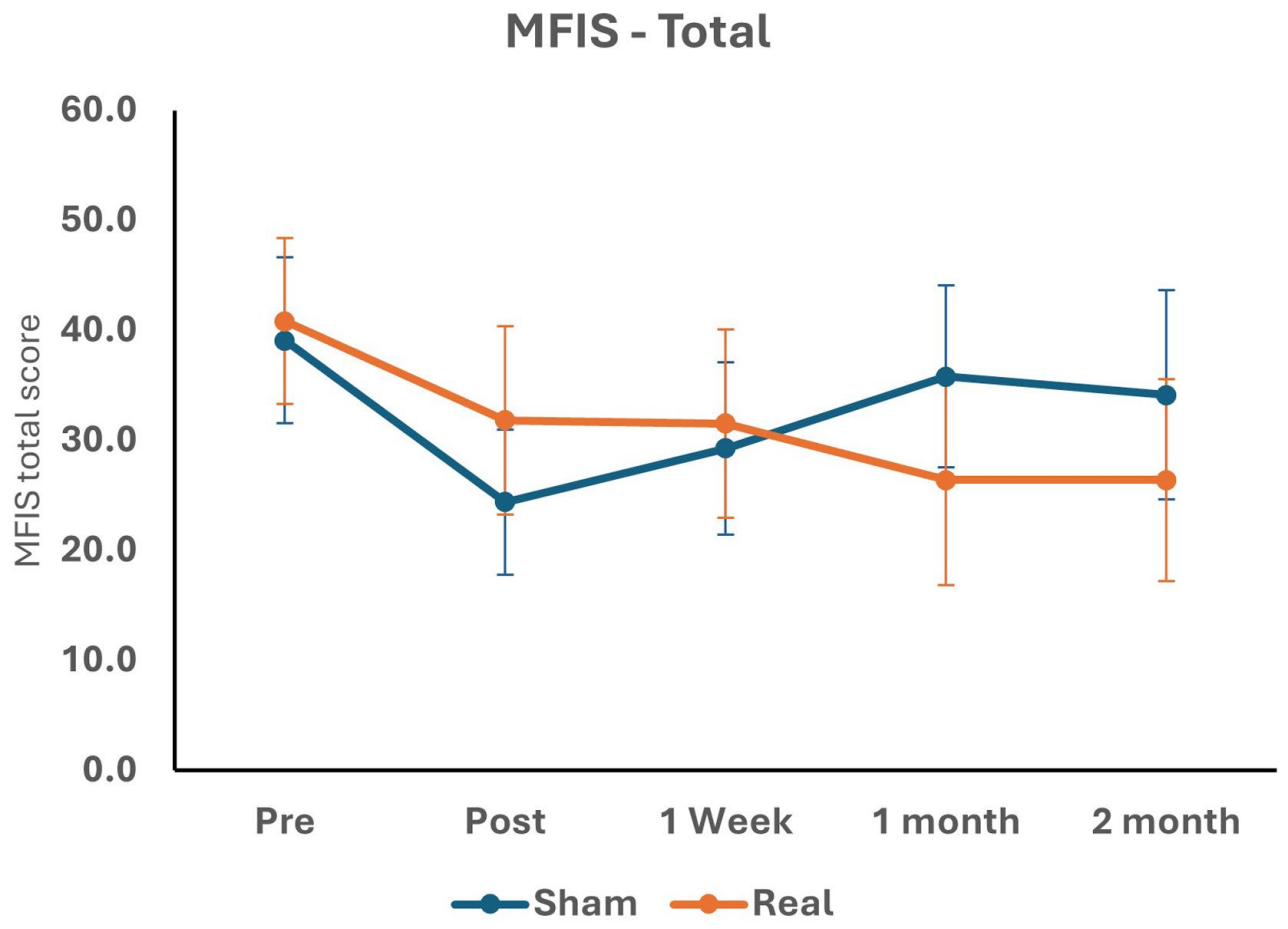
MFIS total score across five study periods.

At T1, there were no significant differences in the MFIS overall (between-group mean difference = 6.00, 95% confidence interval [CI] −4.63 to 16.63, *p* = 0.258), physical (between-group mean difference = 3.11, 95% CI −1.41 to 7.62, *p* = 0.170), cognitive (between-group mean difference = 3.22, 95% CI − 2.47 to 8.91, *p* = 0.256), or psychosocial scores (between-group mean difference = 0.63, 95% CI −0.66 to 1.92, *p* = 0.325) between the active tDCS and sham groups. Similarly, there were no significant differences in the MFIS overall or sub-scores at T2, T3, or T4 between the active tDCS and sham groups (Table 3; Figure 4).

**Table 3:** Outcomes in the active and sham group at various time points.

Outcome	Timepoint	Active group, Mean (SD) *n* = 17	Sham group, Mean (SD) *n* = 17	Between group difference (95% CI)	*p* value^a^
MFIS score ^c^	T0	40.29 (16.33)	38.29 (15.68)	—	
	T1	31.82 (19.16)	24.41 (12.39)	6.00 (−4.63–16.63)	0.258
	T2	31.00 (18.77)	29.29 (16.96)	−4.41 (−15.41–6.60)	0.419
	T3	24.47 (19.74)	31.71 (18.06)	−10.74 (−24.16–2.67)	0.112
	T4^*^	23.71 (18.19)	34.13 (20.36)	−7.70 (−21.51–6.10)	0.262
MFIS Physical score	T0	16.59 (6.35)	17.82 (7.03)	—	
	T1	13.82 (8.67)	11.59 (5.83)	3.11 (−1.41–7.62)	0.170
	T2	13.41 (8.12)	13.94 (6.81)	−1.43 (−5.59–2.73)	0.488
	T3	10.12 (8.30)	14.65 (7.80)	−4.63 (−10.48–1.22)	0.116
	T4^*^	10.94 (8.04)	16.40 (8.78)	−3.28 (−9.46–2.90)	0.286
MFIS Cognitive score	T0	17.82 (8.44)	15.24 (7.17)	—	
	T1	13.59 (8.83)	9.59 (5.99)	3.22 (−2.47–8.91)	0.256
	T2	13.47 (9.25)	11.18 (8.63)	−1.29 (−7.77–5.19)	0.687
	T3	11.35 (9.34)	12.47 (9.83)	−3.97 (−11.36–3.43)	0.282
	T4^*^	9.35 (8.31)	12.73 (10.17)	−3.42 (−10.20–3.36)	0.310
MFIS Psychosocial score	T0	3.47 (2.53)	3.06 (2.49)	—	
	T1	2.35 (2.34)	1.47 (1.55)	0.63 (−0.66–1.92)	0.325
	T2	2.11 (2.29)	2.47 (2.55)	−0.31 (−1.77–1.15)	0.670
	T3	1.65 (1.90)	2.82 (2.24)	−1.18 (−2.67–0.31)	0.115
	T4^*^	1.82 (2.04)	2.73 (2.55)	−0.30 (−2.19–1.60)	0.752
FSS score ^b^	T0	49.18 (6.26)	50.12 (6.30)	—	
	T1	42.47 (9.05)	44.24 (9.32)	−0.92 (−8.33–6.49)	0.802
	T2	44.47 (9.88)	43.53 (12.33)	2.82 (−5.17–10.80)	0.476
	T3	40.29 (9.46)	41.76 (12.11)	0.41 (−7.18–8.00)	0.914
	T4^*^	38.12 (12.97)	44.07 (12.14)	−2.41 (−12.19–7.36)	0.617
PASE score ^d^	T0	86.09 (57.02)	71.26 (60.87)	—	
	T1	93.57 (61.04)	78.56 (66.16)	13.86 (−25.68–35.39)	0.479
	T2	99.09 (77.69)	86.44 (59.90)	0.83 (−37.02–38.67)	0.965
	T3	112.09 (82.84)	99.34 (61.48)	−1.78 (−48.66–45.11)	0.939
	T4^*^	96.27 (64.36)	105.33 (90.64)	−31.03 (−71.90–9.84)	0.131
BI score ^e^	T0	94.12 (10.19)	95.59 (6.35)	—	
	T1	95.00 (9.01)	93.82 (6.26)	3.46 (−0.68–7.60)	0.098
	T2	90.59 (24.68)	94.12 (6.67)	3.37 (−8.5–15.24)	0.566
	T3	93.82 (11.80)	90.59 (12.61)	5.90 (−1.63–13.43)	0.120
	T4^*^	91.18 (18.25)	93.67 (7.43)	−3.36 (−11.81–5.09)	0.422
GDS score ^f^	T0	10.29 (3.46)	8.53 (5.15)	—	
	T1	9.71 (4.01)	8.59 (5.52)	−0.72 (−2.45–1.00)	0.399
	T2	9.41 (4.62)	8.76 (5.91)	−1.87 (−4.12–0.39)	0.102
	T3	7.29 (5.35)	9.06 (6.55)	−3.03 (−6.12–0.06)	0.055
	T4^*^	7.82 (5.67)	9.27 (5.82)	−1.61 (−4.43–1.21)	0.253
HADSA score ^g^	T0	6.59 (4.47)	4.65 (3.79)	—	
	T1	5.24 (4.21)	3.71 (4.44)	−0.06 (−2.83–2.71)	0.968
	T2	4.53 (4.23)	4.18 (3.45)	−0.15 (−3.88–0.88)	0.207
	T3	5.24 (4.49)	4.41 (4.74)	−0.33 (−3.28–2.63)	0.824
	T4^*^	4.41 (5.11)	4.53 (4.17)	−1.85 (−5.09–1.40)	0.253
SSQoL score ^h^	T0	175.41 (38.97)	180.12 (34.44)	—	
	T1	184.82 (32.80)	185.06 (31.20)	8.83 (−2.12–19.79)	0.110
	T2	180.12 (45.97)	182.00 (36.93)	11.16 (−3.75–26.06)	0.137
	T3	189.24 (42.11)	185.35 (38.73)	12.08 (−4.33–28.48)	0.143
	T4^*^	189.94 (44.62)	179.47 (37.50)	9.79 (−8.97–28.55)	0.294

^*^Missing data attributed to one dropout in the sham group at T4.

FFS = Fatigue Severity Scale, MFIS = Modified Fatigue Impact Scale, PASE = Physical Activity Scale for the Elderly, BI = Barthel Index, GDS = Geriatric Depression Scale, HADSA = Hospital Anxiety and Depression Scale – Anxiety, SSQoL = Stroke Specific Quality of Life Scale; T0 = bassline; T1 = end of intervention; T2 = 1-week follow up; T3 = 4-week follow up; T4 = 8-week follow up.

^a^*p*-values for group effect were calibrated by analysis of covariance (ANCOVA) approach, with baseline value, age, and education as covariates.

^b^Possible range for total scores is 9 to 63; higher scores indicate more sever fatigue; a total score of less than 36 suggests that the individual is not experiencing fatigue.

^c^Possible range for total scores 0 to 84; range for physical score is 0 to 36; range for cognitive score is 0 to 40; range for psychosocial score is 0 to 8; higher scores indicate fatigue is more significantly affecting your life.

^d^Possible range for total score is 0 to 400 or more; higher score indicates high level of physical activity in elderly individuals.

^e^Possible range for total score is 0 to 100; higher scores representing greater independence.

^f^Possible range for total score is 0 to 30; Cutoff: normal-0–9; mild depressives-10–19; severe depressives-20–30.

^g^Possible range for total score is 0 to 21; higher scores representing severer anxiety.

^h^Possible range for total score is 49 to 245; higher scores indicate better function.

### Secondary outcomes

The FSS score showed no significant between-group differences across all time points. The mean between-group differences at T1, T2, T3, and T4 were −0.92 (95% CI −8.33 to 6.49, *p* = 0.802), 2.82 (95% CI −5.17 to 10.80, *p* = 0.476), 0.41 (95% CI −7.18 to 8.00, *p* = 0.914), and −2.41 (95% CI −12.19 to 7.36, *p* = 0.617), respectively. The PASE score also showed no significant between-group differences at T1 (mean difference = 13.86, 95% CI −25.68 to 35.39, *p* = 0.479), T2 (mean difference = 0.83, 95% CI −37.02 to 38.67, *p* = 0.965), T3 (mean difference = −1.78, 95% CI −48.66 to 45.11, *p* = 0.939), or T4 (mean difference = −31.03, 95% CI −71.90 to 9.84, *p* = 0.131). Furthermore, the BI score showed no significant between-group differences at T1 (mean difference = 3.46, 95% CI −0.68 to 7.60, *p* = 0.098), T2 (mean difference = 3.37, 95% CI −8.50 to 15.24, *p* = 0.566), T3 (mean difference = 5.90, 95% CI −1.63 to 13.43, *p* = 0.120), or T4 (mean difference = −3.36, 95% CI −11.81 to 5.09, *p* = 0.422). The GDS score showed no significant between-group differences across all time points (T1: mean difference = −0.72, 95% CI −2.45 to 1.00, *p* = 0.399; T2: mean difference = −1.87, 95% CI −4.12 to 0.39, *p* = 0.102; T3: mean difference = −3.03, 95% CI −6.12 to 0.06, *p* = 0.055; and T4: mean difference = −1.61, 95% CI −4.43 to 1.21, *p* = 0.253). The HADSA score showed no significant between-group differences at T1 (mean difference = −0.06, 95% CI −2.83 to 2.71, *p* = 0.968), T2 (mean difference = −0.15, 95% CI −3.88 to 0.88, *p* = 0.207), T3 (mean difference = −0.33, 95% CI −3.28 to 2.63, *p* = 0.824), or T4 (mean difference = −1.85, 95% CI −5.09 to 1.40, *p* = 0.253). Finally, the SSQoL score showed no significant between-group differences at T1 (mean difference = 8.83, 95% CI −2.12 to 19.79, *p* = 0.110), T2 (mean difference = 11.16, 95% CI −3.75 to 26.06, *p* = 0.137), T3 (mean difference = 12.08, 95% CI −4.33 to 28.48, *p* = 0.143), or T4 (mean difference = 9.79, 95% CI −8.97 to 28.55, *p* = 0.294; Table 3).

### Adverse events

Thirty-four participants completed the side-effects survey (17 in the active group and 17 in the sham group). Itching and skin redness were more common in the active group than in the sham group (59% versus 11%, *p* = 0.010). However, the occurrence of other side-effects (tingling, warmth/heat, pinching, fatigue, pain, burning, metallic taste, or headache) was not significantly different between the groups. The majority of these adverse events were rated as mild. In the active tDCS group, one participant reported considerable itching (*n* = 1), two participants reported moderate skin redness (*n* = 2), and one participant reported moderate pinching (*n* = 1). In the active group, discomfort most often appeared at the beginning of stimulation (82%) and subsided by the end (65%). In the sham group, discomfort typically arose around the middle of the stimulation (33%) and resolved quickly (39%). Both groups reported that this discomfort was mostly localized over the head (Table 4).

**Table 4:** Adverse events table.

Adverse events	Active (*n* = 17)	Sham (*n* = 17)	*p*-value ^a^
Itching, *n* (%)	10 (58.8)	2 (11.1)	0.010
Mild	9 (52.9)	2 (11.1)	
Moderate	0 (0)	0 (0)	
Considerable	1 (5.9)	0 (0)	
Strong	0 (0)	0 (0)	
Skin redness, *n* (%)	10 (58.8)	2 (11.1)	0.010
Mild	8 (47.1)	2 (11.1)	
Moderate	2 (11.8)	0 (0)	
Considerable	0 (0)	0 (0)	
Strong	0 (0)	0 (0)	
Tingling, *n* (%)	8 (47.1)	12 (66.7)	0.296
Mild	8 (47.1)	11 (61.1)	
Moderate	0 (0)	1 (5.6)	
Considerable	0 (0)	0 (0)	
Strong	0 (0)	0 (0)	
Warmth/heat, *n* (%)	4 (23.5)	2 (11.1)	0.656
Mild	4 (23.5)	2 (11.1)	
Moderate	0 (0)	0 (0)	
Considerable	0 (0)	0 (0)	
Strong	0 (0)	0 (0)	
Pinching, *n* (%)	4 (23.5)	2 (11.1)	0.656
Mild	3 (17.6)	2 (11.1)	
Moderate	1 (5.9)	0 (0)	
Considerable	0 (0)	0 (0)	
Strong	0 (0)	0 (0)	
Fatigue, *n* (%)	3 (17.6)	5 (27.8)	0.688
Mild	3 (17.6)	3 (16.7)	
Moderate	0 (0)	2 (11.1)	
Considerable	0 (0)	0 (0)	
Strong	0 (0)	0 (0)	
Pain, *n* (%)	1 (5.9)	2 (11.1)	1.000
Mild	1 (5.9)	2 (11.1)	
Moderate	0 (0)	0 (0)	
Considerable	0 (0)	0 (0)	
Strong	0 (0)	0 (0)	
Burning, *n* (%)	1 (5.9)	1 (5.6)	1.000
Mild	1 (5.9)	0 (0)	
Moderate	0 (0)	1 (5.6)	
Considerable	0 (0)	0 (0)	
Strong	0 (0)	0 (0)	
Metallic/iron taste, *n* (%)	0 (0)	1 (5.6)	1.000
Mild	0 (0)	1 (5.6)	
Moderate	0 (0)	0 (0)	
Considerable	0 (0)	0 (0)	
Strong	0 (0)	0 (0)	
Headache, *n* (%)	0 (0)	0 (0)	-
Mild	0 (0)	0 (0)	
Moderate	0 (0)	0 (0)	
Considerable	0 (0)	0 (0)	
Strong	0 (0)	0 (0)	
When did any discomfort begin?			
At the beginning of the stimulation	14 (82.4)	5 (27.8)	0.007
At approximately the middle of the stimulation	3 (17.6)	6 (33.3)	
Toward the end of the stimulation	0	4 (22.2)	
How long did it last?			
It stopped at the end of the stimulation	11 (64.7)	6 (33.3)	0.089
It stopped quickly	6 (35.3)	7 (38.9)	
It stopped in the middle of the stimulation	0	2 (11.1)	
Were the sensations located over the head or in a different location?			
On the head	15 (88.2)	11 (61.1)	0.091
Other: arms	2 (11.8)	3 (16.7)	

^a^Fisher’s exact test.

## Discussion

In a sample of patients with chronic stroke, we tested the effect of tDCS on fatigue symptoms. We found that tDCS did not ameliorate fatigue severity. Existing evidence for the effect of tDCS on PSF is mixed. Gandiga et al. reported that tDCS of the motor cortex had no overt effect on fatigue in patients with chronic stroke (69). Cleland et al. reported that administering two sessions of tDCS per week over the motor cortex for 5–6 weeks in 10 stroke patients did not produce any changes in PSF (26). Ulrichsen et al. reported no add-on effect of six sessions (two times per week) of tDCS (over the left dorsolateral prefrontal cortex, DLPFC) combined with cognitive training on fatigue in 74 patients with chronic stroke (27).

In contrast, two RCTs have shown that tDCS alleviates PSF. In one trial reported by Dong et al., administering six sessions of tDCS per week over the left DLPFC for 4 weeks in 60 stroke patients led to a reduction in fatigue (28). However, there are major differences between the study by Dong et al. and the present study. First, in the former, patients with a duration of stroke of more than 1 year were excluded and the average duration of stroke was only 42 days, whereas in the latter, the average duration was much longer, i.e., 6.4 years. Second, patients with depression were excluded from the former but not from the latter. Third, 24 sessions of stimulation were delivered in the former, while 10 sessions of stimulation were delivered in the latter. Fourth, the anodal and cathodal electrode were placed over the left DLPFC and on the superior margin of the orbit, respectively, in the former, but were placed over the motor cortex and on the arm, respectively, in the latter. Hence, the differences between the results of these two studies may be attributable to differences between their respective participants’ characteristics (duration of stroke and presence or absence of depression or anxiety), number of stimulation sessions, and electrode locations. The second previous RCT was reported by De Doncker et al. and involved 30 patients (20 versus 10) with PSF. It found that compared with sham stimulation, a single session of anodal tDCS over the bilateral primary motor cortex resulted in a reduction in PSF symptoms 1 week later but not 5 weeks later (23). However, the study by De Doncker et al. and the present study differ in terms of the participants’ mean age (64 versus 58 years), duration of stroke (6.4 versus 4.5 years), and the number of sessions of stimulation they received (10 versus 1). In addition, De Doncker et al. included only patients with minimal impairment and excluded patients with depression or anxiety. Hence, the differences between the results of these two studies may be attributable to differences between their respective participants’ characteristics (age, duration of stroke, level of impairment, and presence of absence of depression or anxiety), number of stimulation sessions, and electrode positions.

There are several potential explanations for the lack of efficacy of tDCS in the present study. First, our cohort exhibited chronic stroke, and two recent studies reported that tDCS provided limited or no additional benefits relative to active or sham conditions for PSF in chronic stroke populations (23, 27). Similarly, pharmacological treatments for PSF have shown limited effectiveness in chronic stroke populations, as exemplified by the double-blind, placebo-controlled study by Choi-Kwon et al. that found that fluoxetine did not alleviate fatigue in chronic stroke patients (71). Furthermore, tDCS interventions have been found to be ineffective in chronic stroke patients for other outcomes besides fatigue, such as motor recovery (72) and pain (73).

Second, according to the cut-off scores of the HADSA (52, 53) and the GDS (60, 61), 56% of the participants in the present study had comorbid depression and 15% had concurrent anxiety. Concurrent depression and anxiety may attenuate the ability of tDCS to alleviate PSF. Specifically, the presence of anxiety symptoms has been associated with a blunted response to tDCS; e.g., De Doncker et al. reported that the effects of tDCS on PSF were less pronounced in participants with higher anxiety scores than in those with lower anxiety scores (23). Similarly, Ulrichsen et al. found that tDCS had no significant add-on effects on fatigue in chronic stroke patients who concurrently experienced substantial depressive symptoms, indicating that comorbid depression may reduce responsiveness to tDCS interventions (27). Interestingly, it has also been found that mood may mediate the therapeutic efficacy of tDCS on fatigue caused by stroke or other neurological conditions such as multiple sclerosis (MS) (22, 74). For example, the use of tDCS for fatigue reduction in MS patients has been found to improve their mood (22, 74, 75), suggesting that the effects of tDCS on these outcomes occurs via common neural pathways (74, 75). Finally, comorbid depression may influence the choice of location of an anodal electrode, as discussed in the following paragraph.

Third, stimulation of the motor cortex and the left DLPFC has been found to yield both positive (23, 28) and negative results in stroke patients (26, 27, 69), but there has been no direct comparison of the effects of stimulating each of these sites. In addition, the majority of the participants in the present study had comorbid depression or anxiety, both of which can cause fatigue. Anodal left DLPFC tDCS was previously found to be associated with improved outcomes compared with other-site tDCS in the treatment of depression (76). Similarly, tDCS applied over the DLPFC was found to reduce symptoms of anxiety disorders (77). Conversely, motor cortex stimulation is not known to have an effect on depression or anxiety. Hence, DLPFC stimulation, but not motor cortex stimulation, may decrease fatigue symptoms by alleviating depression and anxiety. This treatment also appears to have no substantial floor or ceiling effects, as none of the participants in the present study achieved the lowest or highest possible scores in the total, physical, and cognitive domains of the MFIS at baseline. In the psychosocial domain, only one (3%) patient received the highest possible score, whereas 6 (17%) patients received the lowest possible score.

Fourth, Vergallito et al. (78) reported significant inter-individual variability in the response to tDCS. Specifically, they found that while tDCS had no effect on excitatory circuits in their whole sample, cluster analysis revealed that it led to a bimodal response pattern, i.e., 50% of the participants responded to tDCS as expected. Possible reasons for this variability in responses to tDCS include differences in cortical thickness, genetics, time of day of stimulation, and coil orientation. In the present study, a cluster analysis of the change in MFIS scores and the original MFIS scores within the active tDCS group revealed one cluster. Hence, there may have been inter-individual variability in our sample.

Fifth, PSF is a heterogeneous syndrome with distinct phenotypes that exhibit distinguishable physical and mood characteristics. For example, a previous clustering analysis identified four distinct subgroups of PSF patients based on laterality of stroke, the level of depression and anxiety symptoms, and motor function (79). Thus, the heterogeneous nature of PSF may contribute to the negative results in tDCS trials aimed at its alleviation (80).

tDCS has been investigated as a potential treatment for fatigue related to other neurological conditions, including MS (81), Parkinson’s disease (82), and post-polio syndrome. For example, several studies involving over 50 MS patients who received five tDCS sessions targeting either the motor cortex (83) or the somatosensory cortex (46, 70) demonstrated a reduction in fatigue symptoms (46, 70, 83), with improvements lasting up to three weeks (83). In another study, 27 MS patients received 20 tDCS sessions over the left DLPFC, resulting in decreased fatigue symptoms (46). Additionally, 17 MS patients treated with 10 sessions of random noise stimulation over the primary motor cortex also experienced fatigue reduction (84). In post-polio fatigue, two studies reported that 10 (85) or 15 (86) daily tDCS sessions significantly lessened fatigue symptoms. A clinical trial involving 23 Parkinson’s disease patients found that eight daily tDCS sessions reduced fatigue as well (87). Importantly, the above-mentioned studies reported only mild side-effects, such as headache and tingling sensations, with no serious side-effects. Three neurophysiological mechanisms have been proposed to underlie central fatigue: slowed conduction in central motor pathways leading to reduced recruitment of spinal motoneurons; conduction block at Ranvier’s nodes; and dysfunction in the prefrontal cortex, which plays a key role in motor planning (83). tDCS may help reduce PSF through multiple pathways. First, it may restore activity in the prefrontal and motor regions (83). Second, it may enhance connectivity between the motor and frontal areas, as well as the thalamus (83). Third, tDCS may improve mood and reduce depressive symptoms (87).

Further studies of tDCS in the treatment of PSF are required. Indeed, there are several ongoing trials on this topic. One of these trials is assessing six sessions of tDCS over the DLPFC combined with routine rehabilitation in 100 patients with PSF (29). Another cross-over trial is determining whether a single session of high-definition tDCS over three different cortical areas (DLPFC, primary motor cortex, and parietal cortex) reduces fatigue symptoms in 20 patients with PSF (88). A third study aims to evaluate the effect of tDCS stimulation of the primary motor cortex combined with virtual reality treatment in 28 patients with chronic stroke (89). In terms of stimulation parameters, future studies should consider a higher current intensity (up to 4.0 mA) (90), a longer duration of stimulation per session (91), and more sessions (up to 24) (29).

This study had several limitations. First, it was a single-center trial. Second, the sample size was small, which reduced the power of the study. A post-hoc power analysis was conducted using G*Power based on an analysis of covariance with two groups (sham versus active tDCS) and five time-points. Assuming a large effect size (*f* = 0.40), an alpha level of 0.05, and a correlation of 0.5 between repeated measurements, our sample size of 34 yielded a power of 62%. Third, the participants were patients with chronic stroke, and therefore, the findings may not be applicable to patients with subacute or acute stroke. Fourth, all of our outcomes were scale evaluations, rather than measurements of objective indicators. Fifth, we did not use an objective measure of motor fatigue. Sixth, the patients did not receive concurrent physiotherapy.

This study provides four implications for future research. First, as PSF is a heterogeneous syndrome, it is unlikely that tDCS at a single site will benefit all PSF patients. Second, careful characterization and selection of a sample that is homogeneous in terms of clinical and imaging features (e.g., in terms of chronicity of stroke) is recommended. Third, comorbid depression and anxiety may influence the choice of tDCS site and the effectiveness of tDCS in alleviating PSF. Fourth, a sample size should be adequate to detect clinically meaningful changes in outcomes, and calculation of the sample size should consider the possibility of inter-individual variability in responses to tDCS.

## Conclusion

We found no evidence that the use of tDCS alleviates PSF. In particular, our findings suggest that motor cortex stimulation may not effectively treat PSF in patients with comorbid depression and/or anxiety. Further research should consider the potential impact of comorbid depression, chronicity of stroke, and selection of a homogeneous group of subjects and an adequate sample size, with consideration of inter-individual variability.

## Data Availability

The raw data supporting the conclusions of this article will be made available by the authors, without undue reservation.
